# Development and validation of the dizziness symptoms questionnaire in Thai-outpatients

**DOI:** 10.1016/j.bjorl.2021.05.007

**Published:** 2021-05-28

**Authors:** Ravin Suvanich, Uraiwan Chatchawan, Chanchai Jariengprasert, Kwanchanok Yimtae, Torkamol Hunsawong, Alongkot Emasithi

**Affiliations:** a Khon Kaen Universiry, Research Center in Back, Neck, Other Joint Pain and Human Performance (BNOJPH), Khon Kaen, Thailand; b Khon Kaen University, Faculty of Associated Medical Sciences, School of Physical Therapy, Khon Kaen, Thailand; c Mahidol University, Faculty of Medicine Ramathibodi Hospital, Department of Otolaryngology, Bangkok, Thailand; d Khon Kaen University, Khon Kaen Ear, Hearing, and Balance Research Center, Faculty of Medicine, Department of Otorhinolaryngology, Khon Kaen, Thailand

**Keywords:** Dizziness, Vestibular symptoms, Questionnaires

## Abstract

**Introduction:**

“Dizziness” is a common complaint in clinical practice that can occur with anyone. However, since the symptom is caused by a wide range of disorders, a general clinician usually faces some difficulty to detect the cause.

**Objective:**

This study aimed to formulate and validate a simple instrument that can be used to screen and predict the most likely cause of dizziness in Thai outpatients.

**Methods:**

This study was divided into two phases. Phase I included 41 patients diagnosed with common causes of dizziness to determine the algorithm and construct the “structural algorithm questionnaire version 1”. In addition, to test and retest its content validity and reliability until the instrument had an acceptable level of both. Phase II of the study pertained to evaluating its accuracy in clinical trials, 150 patients with dizziness had a face-to-face interview while they were waiting for their medical appointment.

**Results:**

The degree of agreement between the algorithm results and clinical diagnoses was within an acceptable level (κ = 0.69). Therefore, this algorithm was used to construct the structural algorithm questionnaire version 1. The content validity of the structural algorithm questionnaire version 1 evaluated by seven experts. The content validity index values of the questionnaire ranged from 0.71 to 1.0. The Cohen’s kappa coefficient (κ) of intra-rater reliability of the structural algorithm questionnaire version 1 was 0.71. In clinical trials, 150 patients with dizziness had a face-to-face interview while they were waiting for their appointment. The overall agreement between their questionnaire responses and final diagnoses by specialists showed a moderate degree of clinical accuracy (κ = 0.55).

**Conclusions:**

The structural algorithm questionnaire version 1 had a well-developed design and acceptable quality pertaining to both validity and reliability. It might be used to differentiate the cause of dizziness between vestibular and non-vestibular disorders, especially of outpatients with dizziness symptoms.

## Highlights

• History taking plays an essential part in evaluating patients with dizziness.
• The algorithm of the SAQ-1 had been developed based on timing-trigger approach.
• The SAQ-1 might be used to triage the cause of dizziness of outpatients.

## Introduction

Dizziness is the second most common complaints in the daily clinical practice, with an estimated lifetime prevalence between 20%–30%.^1^, ^2^ The feeling, however, is quite subjective and possibly originated from numerous disorders including vestibular, cardiovascular, neurologic, metabolic, and psychiatric diseases.^3^, ^4^ Therefore, physicians often face some difficulty in diagnosing the cause of dizziness.^5^ Approximately three out of four patients complaining of dizziness get the correct diagnosis based only on their historical data.^4^, ^6^ History taking plays a crucial part in evaluating patients with dizziness.^4^, ^6^, ^7^, ^8^, ^9^, ^10^, ^11^, ^12^ Nevertheless, it is a difficult task. The meaning of the word “dizziness” itself is ambiguous and covers various sensations such as vertigo, faint, woozy, weak, or unsteady. Many patients have tendency to be uncertain and unreliable^13^ when describing their symptoms, and their complaints usually involve anxiety.^9^ Therefore, to make a differential diagnosis based on their description of symptoms is quite troublesome in primary care settings.^6^ In consequence, patients with dizziness occasionally receive either insufficient or inappropriate diagnosis and treatment.^14^ According to our literature review, some investigators attempted to create the questionnaire based on patients’ symptoms as a differential diagnosis tool. The predictive power ranged from 60% to 84% in those studies.^6^, ^8^, ^12^, ^15^^,^^16^ However, prior studies were formulate with the numbers of self-administration questions (between 4–163 items) and available only in English language.^6^, ^8^, ^12^, ^16^^,^^17^ Differences in language may affect the understanding and responses of patients. Furthermore, there has never been a general available report or publication about using an algorithm of a structured questionnaire.

Therefore, we aimed to create an interview questionnaire, using an algorithm approach to suggest the possible diagnosis of common vestibular disorders in Thai outpatients, and then evaluate its reliability and validity.

## Methods

**Figure 1:**
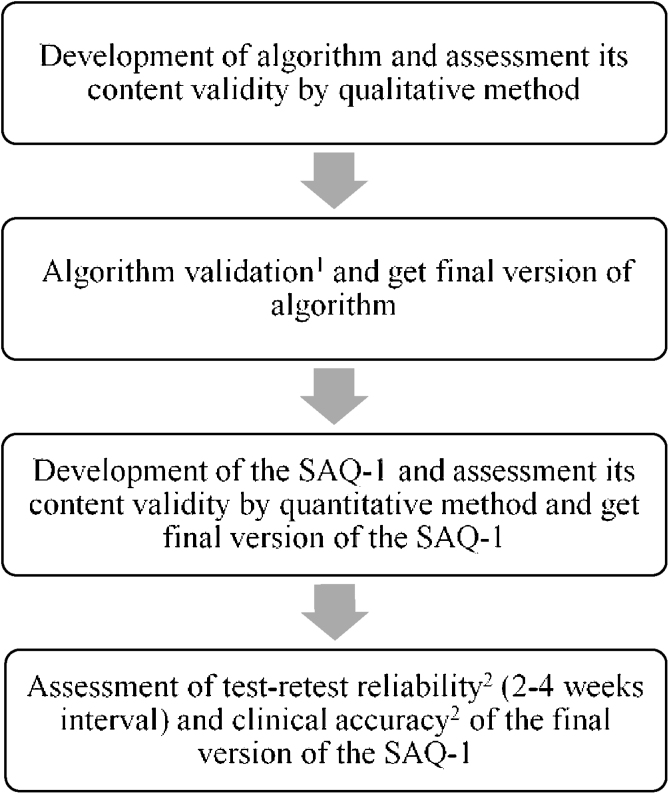
Flowchart represent the process of the study. ^1^Assessed in outpatients who diagnosed with common causes of dizziness. ^2^Assessed in outpatients with new dizziness complaints.

This study was conducted in two tertiary care settings from June 2018 to October 2019. The study protocol was reviewed and approved by the Khon Kaen University Ethics Committee for Human Research (HE601466) and the Committee for Research, Faculty of Medicine Ramathibodi Hospital, Mahidol University (MURA2017/915). All participants were given an explanation of purpose and procedure of the study and gave their written informed consent. Two phases were involved in this study: first, the development of the structural algorithm questionnaire version 1 (SAQ-1); second, the test-retest reliability and diagnostic accuracy of the SAQ-1 were investigated in patients with dizziness (Fig. 1).

### Phase I: Developing of SAQ-1

#### Step I: Algorithmic sequence and face validity

The SAQ-1 is a structural algorithm-based questionnaire, aiming to help the clinicians determine the possible cause of dizziness. Developing the SAQ-1 began with choosing the algorithms based on the significant symptom predictors, which consider value of odd ratios and statistic model, from previous studies.^6^, ^8^, ^16^, ^17^, ^18^, ^19^ The series of binary questions then were formulated in line with the chosen algorithms. The sequence of questions was related to time course, triggering factors, and associated symptoms. The final output of the questionnaire was the hypothetical diagnosis based on the diagnostic criteria formulated by the Committee for Classification of Vestibular Disorders of the Bárány Society. The propriety of the algorithmic sequence and validity of all questions was approved by two highly experienced otoneurologists.

#### Step II: Algorithm validation

To validate the algorithm sequence and its final output of the preliminary version of structural algorithm-based questionnaire, the hypothetical diagnosis from the questionnaire was compared to the clinical diagnosis made by otoneurologists. Forty-one outpatients with known cases of dizziness and vertigo within the past 6 months were recruited for this phase. The sample size estimation was based on previous studies.^20^, ^21^ They were all face-to-face interviewed. At least 75% agreement was set as an acceptable level for algorithm validation.^22^, ^23^

#### Step III: Content validity study

After validating the sequence of algorithm, the content of the questionnaire was evaluated. Seven experts, including of 2 otoneurologists, 3 otolaryngologists, 1 audiologist, and 1 physical therapist with over 10 years of clinical experience were invited to judge the relevance of the SAQ-1, using the content validity index (CVI). They were asked to rate the relevance of each item based on a 4-point ordinal scale (1 = not relevant; 2 = somewhat relevant; 3 = relevant; 4 = highly relevant). The relevance of each item in SAQ-1 questionnaire (item‐content validity index [I‐CVI]) as well as the relevance of the entire SAQ‐1 questionnaire (scale‐content validity index [S‐CVI]) were calculated. The acceptable value of the I-CVIs and S-CVIs were at least 0.78 and 0.90, respectively.^24^, ^25^, ^26^, ^27^ Items with values less than the I-CVI threshold of 0.78 were revised based on the expert discussions.

### Phase II: Test–retest reliability and clinical accuracy

To determine the reproducibility and the clinical accuracy of the SAQ-1, the hypothetical diagnosis from the questionnaire was compared between two visits, and with the clinical diagnosis made by experienced otoneurologists at 3-month after the initial visit.

### Participants

Thai patients aged between 18 and 65 years with their first and recent dizziness or vertigo attack within the past 6 months were recruited from the outpatient otolaryngology clinic at the two tertiary care hospitals: Ramathibodi Hospital and Srinagarind Hospital. They had to be able to understand and communicate in Thai language and had to agree to participate in the study. Those who had aphasia, severe cognitive impairment and mental disabilities, or other conditions that could impair their ability to participate in the interviewing process were excluded.

### Procedures

On their first visit, the eligible participants were interviewed by trained interviewers using the SAQ-1 before their medical appointments. Then, they were asked to be interviewed again during their follow-up visit, scheduled in two to four weeks. To investigate the diagnostic accuracy of the SAQ-1, the hypothetical diagnosis from the SAQ-1 was later compared to the diagnosis made by 2 experienced otoneurologists. They also followed the diagnostic criteria based on the committee for classification of vestibular disorders of the Bárány Society.

### Statistical analyses

The degree of agreement between the algorithm results and the final diagnosis in Step II of Phase I used proportion agreement and Cohen’s kappa statistic and a 95% confidence interval.

In Phase II, descriptive statistics was used to describe the demographic characteristic of the participants and to explore the distribution of diagnoses. Cohen’s kappa analysis and a 95% confidence interval were used to determine the reliability of the SAQ-1 and the degree of agreement between the diagnoses originated from the questionnaire results and those made by experienced otoneurologists (clinical accuracy).

All data were analyzed by using the Stata statistical software version 11 program.

## Results

### Phase I: Developing of SAQ-1

#### Step I: Algorithmic sequence and face validity

The preliminary version of structural algorithm-based questionnaire had a total of 20 questions in three domains: 3 in time course of dizziness and vertigo symptoms, 7 in triggering factors, and 10 in associated symptoms. The first question was whether the time course of dizziness was episodic or continuous. The following questions would vary based on the answers to the previous question. The sequence of the algorithm was designed to suggest a hypothetical diagnosis. The minimum number of questions to obtain the diagnosis was 3 while the maximum number was 10. The average time for an interview was 15 min. The structural algorithm-based questionnaire allocated patients into vestibular, or non-vestibular groups. The vestibular group was subsequently divided into benign paroxysmal postural vertigo (BPPV), Meniere’s disease (MD), vestibular migraine (VM), acute unilateral vestibulopathy, and other vestibular disorders. The non-vestibular group included vertebrobasilar insufficiency (VBI), transient ischemic attack (TIA), stroke, Parkinson's disease (PD), ataxia, postural hypotension, cardiovascular disease, ototoxicity, persistent postural-perceptual dizziness (PPPD), and multifactor dizziness.

#### Step II: Algorithm validation

Forty-one outpatients (female/male = 28/13 and age = 49.34 ± 9.68 year) were recruited. Accordingly, they were diagnosed with common causes of dizziness, n (%): BPPV = 11 (26.83); MD = 9 (21.95); VM = 14 (34.15); acute unilateral vestibulopathy = 3 (7.32); other peripheral vestibular = 2 (4.88) and non-vestibular = 2 (4.88).

To validate the algorithm, when comparing the specific vestibular disorders between medical diagnosis made by experienced otoneurologists and the results from the second draft of the algorithm, the agreement was 75.61% with Cohen’s kappa coefficient = 0.69 (*p* < 0.05).

#### Step III: Content validity study

The content validity of the SAQ-1 (20 items) showed that the I-CVI scores ranged from 0.71 to 1.00. Only 4 questions (20%) regarding triggers and associated symptoms needed revision as the I-CVI scores were less than 0.78. Finally, the S-CVI/Ave for the final version SAQ-1 was 0.86. Table 1 presents the final version of the SAQ-1.

**Table 1:** The final version of the SAQ-1.

Item	Details	Answer	
Q1	Episodic attack	□ Yes	□ No
Q2	Single attack	□ Yes	□ No
Q3	Chronic dizziness	□ Yes	□ No
Q4	Triggered by specific head movement: lying down, rolling over, bending over, looking up	□ Yes	□ No
Q5	Triggered by changing to an upright position: lying-to-sitting, sitting-to-standing	□ Yes	□ No
Q6	Triggered by non-specific or all positions of head movements	□ Yes	□ No
Q7	Triggered by pressure change: e.g., cough-sneeze, heavy lifting, Valsava, fast elevators, airplanes, scuba diving, loud sounds	□ Yes	□ No
Q8	Occurred after the trauma onset	□ Yes	□ No
Q9	Occurred after a change in medication: antibiotics, drugs for hypertension, diabetes mellitus, dyslipidemia, arrhythmia, anticonvulsants	□ Yes	□ No
Q10	Occurred after infection onset: fever, headache, ear pain	□ Yes	□ No
Q11	Blackouts or fainting when dizzy	□ Yes	□ No
Q12	Associated with neurologic symptoms	□ Yes	□ No
Q13	Associated with otologic symptoms: hearing loss, tinnitus, ear fullness	□ Yes	□ No
Q14	Associated with fluctuations in hearing loss, tinnitus, ear fullness	□ Yes	□ No
Q15	Associated with sudden unilateral hearing loss	□ Yes	□ No
Q16	Associated with progressive unilateral hearing loss	□ Yes	□ No
Q17	Associated with cervical problems: neck pain, limited movements, arthritis	□ Yes	□ No
Q18	Associated with migraine symptoms	□ Yes	□ No
Q19	Associated with cardiovascular symptoms	□ Yes	□ No
Q20	Associated with stress, anxiety, or certain situations	□ Yes	□ No

### Phase II: Test–retest reliability and clinical accuracy

A total of 173 patients with dizziness complaints were asked to participate in the study. Twenty-three patients refused to participate. The mean age at initial visit was 52.4 ± 10.2 years (range, 25–65 years), with 70% women and 30% men. Table 2 showed the demographic characteristics and diagnoses of all patients.

**Table 2:** Participant characteristic (outpatients with new dizziness complaints) and diagnose’s in phase II study (n = 150).

Characteristics and diagnoses	Total
A) Characteristic	
Age, mean ± SD (years)	52.4 ± 10.2
Gender, n (%)	
Male	45 (30.0)
Female	105 (70.0)
Education, n (%)	
Primary school or lower	16 (10.7)
High school	16 (10.7)
Diploma	18 (12.0)
Bachelor’s degree	67 (44.7)
Master’s degree or higher	32 (21.3)
Other	1 (0.7)
Duration between visits, mean ± SD (days)	24.8 ± 12.9
B) Diagnostic categories, n (%)	
BPPV	39 (26.0)
MD	14 (9.3)
VM	23 (15.3)
Acute unilateral vestibulopathy	2 (1.3)
Other vestibular	28 (18.7)
Non-vestibular	31 (20.7)
Inconclusive	13 (8.7)

BPPV, benign paroxysmal postural vertigo; MD, Meniere’s disease; VM, vestibular migraine; SD, standard deviation.

For the test-retest reliability, 121 patients (80.67%) completed the two visits. The overall percent of agreement of the questionnaire responses between the two visits was 77.70% and Cohen’s kappa coefficient was 0.71 (*p* < 0.05), which indicated substantial agreement (Table 3).

**Table 3:** Test–retest reliability, degree of agreement of the specific questionnaire responses between the initial and follow-up visits (n = 121).

Second visit results (n)	First visit results (n)						
	BPPV	MD	VM	Acute unilateral vestibulopathy	Other vestibular	Non-vestibular disorders	Total
BPPV	26	1	1	0	0	3	31
MD	0	7	2	0	0	0	9
VM	0	2	19	0	3	2	26
Acute unilateral vestibulopathy	0	0	0	2	0	0	2
Other vestibular	0	2	1	0	11	4	18
Non-vestibular disorders	1	1	1	0	3	29	35
Total	27	13	24	2	17	38	121

Percent agreement (95% CI) = 77.70% (70.16–85.21).

Cohen’s Kappa (95% CI) = 0.71 (0.62–0.81).

BPPV, benign paroxysmal postural vertigo; MD, Meniere’s disease; VM, vestibular migraine; CI, confidence interval.

Validation of the final version of SAQ-1 using the initial visit with the “reference standard” of this study, was obtained with the final clinical diagnosis made by experienced otoneurologists at 3-month after the first visit. According to the results, 13 patients had an inconclusive diagnosis, therefore, there were 137 from the total of 150 patients (91.33%) that remained for the analysis of the study accuracy. The final diagnosis of dizziness was broadly categorized into 106 (77.37%) vestibular disorders, and 31 (22.63%) non-vestibular disorders. The final diagnoses and baseline characteristics of these patients are in Table 4. The overall agreement of questionnaire was 64.23% and Cohen’s kappa coefficient was 0.55 (*p* < 0.05). BPPV had the highest percent of agreement, followed by VM, and non-vestibular disorders.

**Table 4:** Clinical accuracy, degree of agreement of the specific questionnaire responses (first visit) and the clinical diagnoses made by experienced otoneurologists of newly diagnosed patients (n = 137).

Questionnaire results (n)	Clinical diagnosis (n)						
	BPPV	MD	VM	Acute unilateral vestibulopathy	Other vestibular	Non-vestibular disorders	Total
BPPV	36	0	0	0	2	1	39
MD	1	5	1	0	5	1	13
VM	1	3	17	0	3	3	27
Acute unilateral vestibulopathy	0	0	0	1	0	1	2
Other vestibular	0	3	2	1	7	3	16
Non-vestibular disorders	1	3	3	0	11	22	40
Total	39	14	23	2	28	31	137

BPPV, benign paroxysmal postural vertigo; MD, Meniere’s disease; VM, vestibular migraine; CI, confidence interval.

## Discussion

History taking is the first and important step to evaluate those patients with dizziness complaints. Traditionally, the quality of symptoms such as dizziness, vertigo, or lightheaded was mainly in focus.^28^ This approach, however, was not practical as these symptoms are not specific and may arise from either vestibular or non-vestibular conditions. Then, the timing-trigger approach was proposed and become widely popular. This approach has an advantage over the traditional approach because patients could explain the characteristics of their symptoms more clearly and accurately.^29^, ^30^, ^31^ The algorithmic sequence of the SAQ-1 was also based on the timing-trigger approach.^32^, ^33^, ^34^ As the history taking is an essential part for diagnostic process, the SAQ-1 aims to help the history taking process to be more systematic, less time consuming, and to attain to a hypothetical diagnosis in the first visit. Several questionnaires have been developed over the years.^6^, ^8^, ^15^, ^16^, ^17^, ^19^ The number of questions ranged from 4 to 163. All previous questionnaires were completed by the patients themselves.^6^, ^8^, ^15^, ^16^, ^17^, ^19^ They would read the questions individually, choosing the answer that would best fit their problem. The SAQ-1, however, consists of scripted interview questions. The interviewer reads each question to them, and they only have 2 choices of answer: yes or no. Therefore, if they do not clearly understand any questions, they can ask for further explanation. A supplementary explanation will be then, read to them. The time taken for an interview lasts no longer than 15 min. Depending upon the algorithm, some will reach the diagnosis after 3 questions while some would need 10.

The clinical accuracy of the SAQ-1 was satisfying. The percentage of agreement between the hypothetical diagnosis from the questionnaire and medical diagnosis from otoneurologists was relatively substantial. However, there were 10% of participants who did not reach their final diagnosis or had inconclusive diagnosis. According to results of the SAQ-1, the possible diagnosis of five subjects were peripheral vestibulopathy such as Meniere’s disease or recurrent vestibulopathy, six subjects were vestibular migraine, and two subjects were non-vestibular causes such as PPPD. The ability of the SAQ-1 to detect the vestibular group is quite great. The ability to differentiate within the vestibular group, however, still needs some improvement. The reason for this fact is that some vestibular disorders have very similar symptoms and somewhat fluctuating.^35^ Vestibular migraine and MD occurs both in episodic attacks, with some overlapping symptoms. As far as we concern, the SAQ-1 adequately serves the purpose of being a screening questionnaire. To obtain the final diagnosis, vestibular function tests is still necessary.

Although the study has reached its aim, some inevitable limitations should be noted. First, the sample size was smaller than what we expected. This is partially due to the approximately 20% drop out and time limit. It is common that patients with dizziness do not come back for follow up when the symptoms are solved. Second, the SAQ-1 renders only single diagnosis. In reality, many patients with dizziness likely experience multifactor conditions. The patients will report only their dominant symptoms.

## Conclusions

In summary, the SAQ-1 has a well-developed design and acceptable quality on both validity and reliability. It helps physicians differentiate the cause of dizziness between vestibular and non-vestibular disorders, especially of outpatients with non-acute, chronic, or recurrent vestibular symptoms. Further study should be conducted to test the performance of this instrument in different clinical settings, especially in primary care settings.

## Funding

This work was supported by the Research Center on the Back, Neck, Other Joint Pain and Human Performance (BNOJPH), Faculty of Associated Medical Sciences, Khon Kaen University.

## Conflicts of interest

The authors declare no conflicts of interest.
