# Supplementary figures and images for: Development and validation of the dizziness symptoms questionnaire in Thai-outpatients
Source: Braz J Otorhinolaryngol. 2021 May 28;88(5):780–6. doi: 10.1016/j.bjorl.2021.05.007 (PMC9483993; doi:10.1016/j.bjorl.2021.05.007)

**BJORL-D-20-00776 - SUPPLEMENTARY MATERIAL**

**Appendix 1** The final version of SAQ-1 algorithm.

**
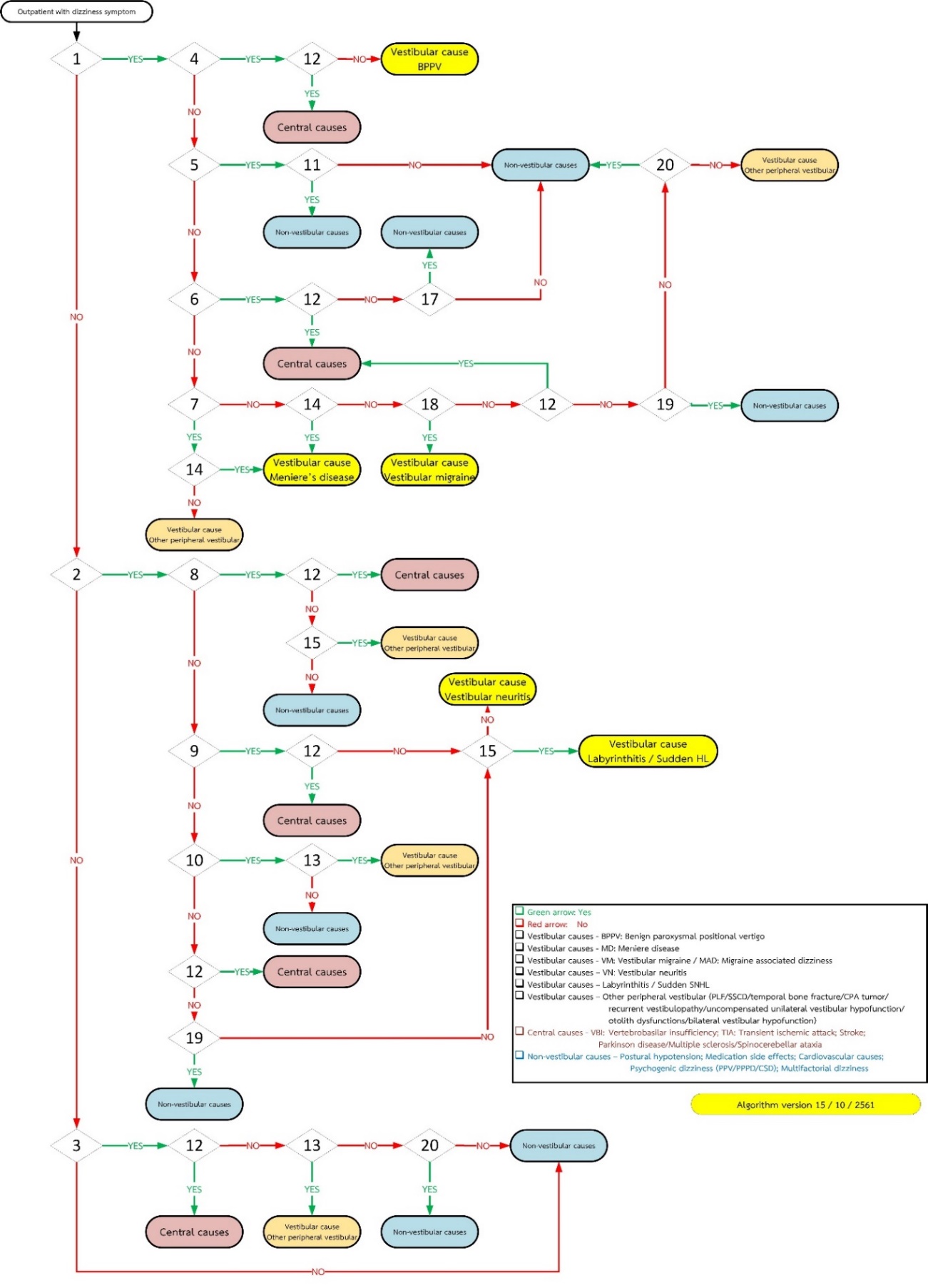
**

Supplement: Supplementary file 1 [file mmc1.docx]
